# Perceptual Temporal Structure Supports Rhythm Learning and Enhances Theta Oscillations When Perception and Action Are Dissociated

**DOI:** 10.3390/brainsci16050489

**Published:** 2026-04-30

**Authors:** Xue Weng, Yang Lu, Xinyue Zhao, Haoran Jiang, Lin Li, Xiuyan Guo

**Affiliations:** 1 School of Psychology and Cognitive Science, East China Normal University, Shanghai 200062, China; wengx0130@hotmail.com (X.W.); tenney.xyzhao@gmail.com (X.Z.); 2 Fudan Institute on Ageing, Fudan University, Shanghai 200433, China; yang_lu@fudan.edu.cn (Y.L.);; 3 Ministry of Education (MOE) Laboratory for National Development and Intelligent Governance, Fudan University, Shanghai 200433, China; 4 Silver-X MOE Philosophy & Social Sciences Laboratory, Fudan University, Shanghai 200433, China

**Keywords:** rhythm learning, perceptual temporal structure, motor timing, theta oscillations, implicit learning

## Abstract

**Highlights:**

**What are the main findings?**

- Rhythm learning primarily relies on perceptual temporal input rather than motor execution alone.
- Global theta oscillations are selectively enhanced during successful perceptual rhythm learning and index unconscious knowledge acquisition.

**What are the implications of the main findings?**

- The paradigm enables independent manipulation of perceptual and motor rhythms, providing a new approach to studying sequence learning.
- Perceptually driven rhythmic knowledge can be acquired implicitly and outperforms motor execution alone, with global theta oscillations as a key neural signature.

**Abstract:**

Background: Rhythmic knowledge enables the precise timing of actions in dynamic environments. Although rhythm learning has been extensively studied, it remains debated whether such learning arises primarily from the perceptual encoding of rhythmic inputs or from the repetitive execution of periodic actions. Methods: To address this question, we developed a temporal-rhythm serial reaction time (TR-SRT) paradigm that dissociates rhythmic structures in perceptual inputs from the timing of motor responses. Across three experiments, participants learned rhythms under visuomotor (Experiment 1, N = 27), visual-only (Experiment 2, N = 26), or motor-only (Experiment 3, N = 26) conditions while electroencephalography was recorded. Results: Behavioral learning slopes revealed robust rhythm learning in both the visuomotor and visual-only conditions, whereas no learning emerged when rhythmic structure was confined to motor timing alone. Post-learning awareness tests further indicated that the acquired rhythmic knowledge was predominantly implicit. Consistently, global (whole-brain) theta-band magnitude (4.8–5.2 Hz) was enhanced only in the conditions that supported rhythm learning. Conclusions: These findings indicate that rhythm learning depends primarily on perceptual temporal structure rather than the repetition of rhythmic actions and identify increased global theta oscillations as a neural signature of this perceptually driven and largely implicit learning process.

## 1. Introduction

Temporal structures pervade the human environment and guide perception, prediction, and coordinated action. Learning such regularities enables individuals to anticipate event timing and improves performance in behaviors such as speech, music, and skilled movement. Temporal structure has been identified as a key source of predictive processing [1], and rhythm has long been recognized as a foundational element of perceptual–motor coordination [2,3,4,5,6,7,8].

Despite extensive research, a fundamental question remains unresolved: Is rhythmic knowledge acquired primarily through perceptual encoding of temporal structure, or through the repeated execution of temporally patterned actions? This question remains difficult to resolve because traditional experimental paradigms inherently couple perceptual and motor rhythms.

The serial reaction time (SRT; [9]) task has been widely used to investigate the implicit learning of temporal and spatial structures [10]. Findings from this paradigm have given rise to two competing accounts of rhythm learning [4]. One perspective proposes that rhythmic knowledge is acquired through perceptual encoding of temporal regularities. Studies using passive observation or sensory-based tasks show that individuals can extract temporal patterns from visual or auditory sequences even without performing overt motor responses [11,12,13]. Additional evidence indicates that perceptual processing alone can support the learning of temporal structure, with attentional and sensory mechanisms contributing to the formation of internal temporal predictions [7,14,15].

A contrasting perspective argues that rhythm learning is primarily driven by the repeated execution of temporally structured actions. Motor-based accounts suggest that internal timing models emerge through practice-dependent motor processes [16,17,18,19]. According to this view, rhythm learning reflects the gradual calibration of motor routines rather than the extraction of perceptual regularities from sensory input. For example, evidence from studies of temporal and spatial sequence learning suggests that motor execution can independently give rise to rhythm acquisition, even when perceptual cues are minimized [20,21,22].

However, a key methodological limitation prevents a decisive test of these accounts. In traditional SRT paradigms, motor responses are typically time-locked to stimulus onset, resulting in an inherent coupling between perceptual and motor rhythms [9,10]. Because the timing of sensory events and actions covary naturally, learning effects cannot be uniquely attributed to perceptual or motor contributions. Efforts to disentangle these influences through passive observation paradigms have produced mixed or inconclusive evidence [18,20,23,24]. Concerns have also been raised regarding the possibility of covert motor imagery during passive tasks, as well as challenges in ensuring adequate attentional engagement [14].

Another limitation of previous work concerns the role of awareness. Although rhythm learning is often assumed to be largely implicit [25,26], few studies have systematically examined whether individuals have conscious access [27]. Methods for distinguishing implicit from explicit structural knowledge—such as those proposed in research on artificial grammar learning and sequence learning—have not been widely implemented in rhythm-learning paradigms [28,29,30].

Beyond behavioral evidence, neural oscillations have increasingly been examined as potential signatures of temporal learning. Theta-band activity (approximately 4–7 Hz) has been consistently linked with the encoding of new temporal and sequential information, as demonstrated across studies in developmental learning, perceptual prediction, and implicit sequence acquisition [31,32,33,34,35,36]. Recent work further suggests that theta oscillations may reflect the formation of internal temporal models that support predictive timing [27,37]. However, whether theta activity specifically indexes the unconscious acquisition of rhythmic structure when perceptual and motor rhythms are experimentally dissociated has not yet been established.

To address these gaps, the present research employs a time-rhythm serial reaction time (TR-SRT) paradigm designed to dissociate perceptual and motor rhythms by independently manipulating stimulus durations and action-locked intervals. This approach allows for perceptual and motor rhythmic structures to be examined separately, overcoming the inherent coupling found in traditional paradigms. Using this paradigm, three experiments were conducted to investigate whether rhythm learning depends on perceptual input, motor execution, or a combination of both. Behavioral learning was assessed through reaction time measures, neural responses were examined through theta-band oscillatory activity, and awareness was evaluated using a structural knowledge test [38] capable of distinguishing implicit from explicit acquisition [29,39].

Together, these experiments provide a systematic test of the perceptual and motor mechanisms underlying rhythm learning and clarify how rhythmic structure is encoded and represented at behavioral and neural levels.

## 2. Materials and Methods

### 2.1. Participants

Across the three experiments, independent samples of healthy young adults were recruited from local universities. All participants had normal or corrected-to-normal vision, were right-handed, and reported no history of neurological or psychiatric disorders. They were naive to the purpose of the study and had no prior experience with implicit learning tasks. To reduce between-participant variability in learning and EEG activity, participants were instructed to maintain a normal night of sleep and to avoid caffeine-containing beverages for at least 12 h prior to the experiment. Written informed consent was obtained in accordance with the institutional ethics guidelines, and participants received monetary compensation for their time. All experiments were approved by the institutional Committee on Human Research Protection.

Sample size estimation was informed by a prior temporal-sequence learning study using a five-item structured sequence comparable to the rhythmic pattern implemented in the present TR-SRT paradigm [40]. That study reported a learning effect of approximately 25.8 ms after 72 sequence presentations. For the current design, in which each session contained 18 rhythmic sequences, this corresponded to an estimated learning slope of approximately 6.4 ms per session (25.8 × 18/72). The reported standard error was 2.56 ms/session with N = 20 participants. Based on these values, a sample size of approximately 25 participants was considered sufficient for the Bayesian evaluation of rhythm learning: if the true slope were around 6 ms/session (H1), the Bayes factor would be expected to exceed 6, whereas if the slope were 0 ms/session (H0), the Bayes factor would be expected to fall below 0.33.

In Experiment 1, twenty-seven participants were recruited (14 males; mean age = 20.78 years, SD = 0.40). One participant was excluded due to excessive EEG artifacts, resulting in a final sample of 26 participants. In Experiment 2, twenty-six participants were recruited (13 males; mean age = 21.77 years, SD = 0.37), and one participant was excluded because approximately 50% of event markers were missing during EEG acquisition, yielding 25 valid participants. In Experiment 3, twenty-six participants were recruited (13 males; mean age = 21.50 years, SD = 0.47), and all contributed valid behavioral and EEG data. These sample sizes satisfied the Bayesian sensitivity requirements outlined above.

### 2.2. Procedure

#### 2.2.1. TR-SRT Task Structure

**Figure 1:**
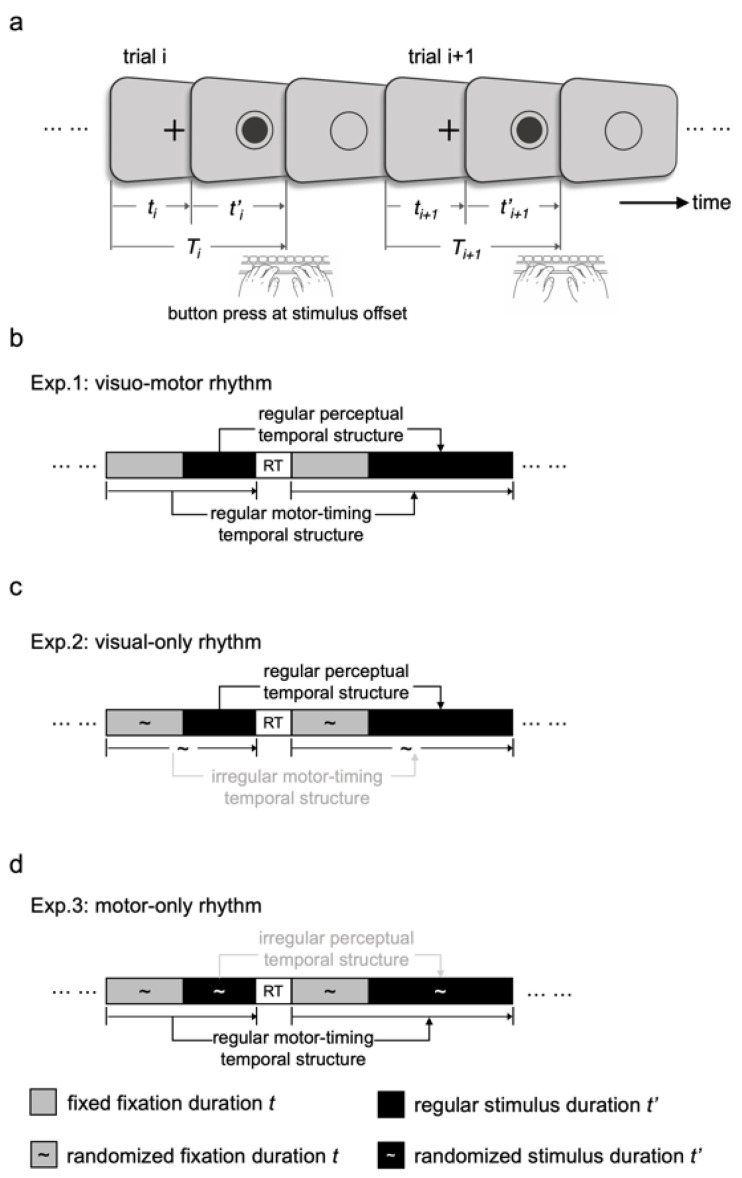
Trial-to-trial structure of time-rhythm serial reaction time (TR-SRT) task. (**a**) Schematic illustration of any two consecutive trials. *t* denotes fixation duration, *t*′ denotes dot-stimulus duration, and *T* = *t* + *t*′ represents the interval from the previous response to the next stimulus offset, which determines the timing of the next response. (**b**–**d**) Schematic logic of the dissociative manipulation of perceptual and motor rhythms in Experiments 1–3. Grey and black squares represent fixation (“+”) and dot-stimulus durations, respectively. The tilde symbol (~) indicates randomized durations in Experiment 2 and pseudo-randomized durations in Experiment 3.

Stimuli were presented on a 1920 × 1080 monitor (60 Hz refresh rate), and participants were seated approximately 70 cm from the screen. Each trial comprised three sequential phases: fixation, stimulus, and response (Figure 1a). A fixation cross appeared at the center of the screen for a duration *t*, followed by a filled dot (visual angle = 1.62°) presented within an unfilled circle (visual angle = 3.20°). After a stimulus duration *t*′, the dot disappeared while the circle remained on the screen. Participants were instructed to press the response button as soon as the dot disappeared within an 800 ms response deadline. Immediately after the button press, the remaining circle disappeared, and the next trial began.

The TR-SRT task consisted of six sessions of 120 trials each. Within each session, 90 trials formed rhythmic sequences (18 five-trial sequences), and 30 trials were random (deviant) trials inserted between successive sequences. Random trials were inserted with 1–4 deviant trials occurring within each inter-sequence gap.

In deviant trials, temporal parameters were pseudo-randomly generated according to the constraints specific to each experiment, while ensuring that no deviant trial had the same stimulus duration as either of its immediately adjacent trials. This procedure prevented the accidental formation of rhythmic patterns within deviant segments.

Participants first completed 60 practice trials composed only of random timing. Practice was repeated until anticipatory or missed responses were below 10%. A one-minute break was provided between sessions. After completing the task, participants were asked whether they perceived any rhythmic structure in the stimulus timing or in their own key presses and were invited to describe it if applicable.

#### 2.2.2. Experiment-Specific Rhythmic Manipulations

Experiment 1: Visuo-Motor rhythm. Rhythmic structure was present in both perceptual input and motor output. Within rhythmic sequences, stimulus durations followed a fixed five-item temporal pattern (200 → 1000 → 600 → 400 → 800 ms). Because the fixation duration remained constant, this structure produced corresponding rhythmic inter-response intervals (IRI: 1000 → 1800 → 1400 → 1200 → 1600 ms).

The first stimulus duration of each rhythmic sequence was randomly sampled from the set {200, 400, 600, 800, 1000 ms}, ensuring equal probability for sequences beginning at different positions within the five-item cycle (Figure 1b).

In deviant trials, stimulus durations were pseudo-randomized while respecting the constraints described in Section 2.2.1.

Experiment 2: Visual-only rhythm. In Experiment 2, participants performed the same key press response task as in Experiment 1. However, rhythmic structure was preserved only in the visual stimulus stream, whereas motor timing was rendered non-rhythmic. Specifically, stimulus durations (*t′ᵢ*) followed the same fixed five-item pattern as in Experiment 1 (200 → 1000 → 600 → 400 → 800 ms), while fixation durations (t) were jittered uniformly between 600 and 1000 ms on every trial. Because the timing of the key press depended on the sum of fixation and stimulus durations (*Tᵢ* = *tᵢ* + *t′ᵢ*), this jitter disrupted regularity in inter-response intervals while leaving the rhythmic structure in the visual stream intact (Figure 1c).

Deviant trials were generated in the same manner as in Experiment 1, except that the fixation durations remained jittered between 600 and 1000 ms.

Experiment 3: Motor-only rhythm. Experiment 3 preserved rhythmic structure in motor timing while eliminating rhythmic structure in the visual stimulus stream. Both fixation durations (*t*) and stimulus durations (*t′*) were pseudo-randomized between 200 and 1300 ms within rhythmic sequences, thereby removing periodicity from the stimulus stream itself. However, the combined interval determining response timing (*Tᵢ* = *tᵢ* + *t′ᵢ*) followed the same fixed five-item pattern used in Experiment 1 (1000 → 1800 → 1400 → 1200 → 1600 ms). This manipulation produced rhythmic regularity exclusively in motor output while maintaining non-rhythmic perceptual timing (Figure 1d).

In deviant trials, fixation and stimulus durations were randomized so that the combined duration (Tᵢ) contained no repeating temporal structure.

#### 2.2.3. Structural Knowledge Test

To assess whether participants had conscious access to the temporal structure acquired during TR-SRT learning, a post-task structural knowledge test was administered immediately after the learning phase in Experiments 2 and 3, following the established procedures in implicit learning research (e.g., [28,29,30,38,39,41,42]). The test comprised 100 trials, each presenting a two-interval chunk. In each trial, participants judged whether the interval pair conformed to the learned temporal structure (“conforming” vs. “nonconforming”), and then reported the subjective basis of their judgment by selecting one of four attributions:

Guess—a response made without any basis, similar to random guessing.

Intuition—a response accompanied by a feeling of confidence but without awareness of the underlying reason.

Memory—a response based on the explicit recollection of encountering (or not encountering) such stimulus pairs during the TR-SRT task.

Rules—a response made because the participant had consciously discovered the underlying temporal rule and could explicitly report it.

Definitions of these categories were displayed continuously on the screen alongside the response options.

In Experiment 2, the test probed awareness of the visual temporal structure. Conforming trials (50/100) consisted of adjacent pairs from the five-item duration pattern: 200 → 1000, 1000 → 600, 600 → 400, 400 → 800, and 800 → 200 ms (*t′*_1_ → *t′*_2_ … *t′*_5_ → *t′*_1_). Nonconforming trials consisted of duration pairs that did not correspond to any adjacent transitions in the rhythmic pattern. Fixation durations were jittered between 600–1000 ms, consistent with the learning task.

In Experiment 3, the test assessed awareness of the motor-timing structure expressed in the combined interval *T*ᵢ = *t*ᵢ + *t*′ᵢ. Conforming trials consisted of adjacent pairs from the five-item *T* pattern: 1000 → 1800, 1800 → 1400, 1400 → 1200, 1200 → 1600, and 1600 → 1000 ms (*T*_1_ → *T*_2_ … *T*_5_ → *T*_1_). Nonconforming trials consisted of interval pairs lacking this temporal structure. The response procedure and attribution categories were identical to Experiment 2, and the participants judged whether the overall timing of the interval pair matched the temporal structure experienced during the preceding TR-SRT task.

### 2.3. EEG Recording and Preprocessing

EEG data were recorded from 64 scalp electrodes arranged according to the 10–20 system. Signals were acquired using an Eego amplifier (ANT Neuro bv, Hengelo, The Netherlands) in Experiment 1 and a Brain Products system (Brain Products GmbH, Gilching, Germany) in Experiments 2 and 3. The two systems were used due to equipment availability at the time of data collection. Previous validation work has suggested that ANT and Brain Products systems can acquire statistically comparable, high-quality EEG signals [43].

All EEG data were processed using an identical preprocessing and analysis pipeline. Across the experiments, EEG was sampled at 1000 Hz with a recording bandpass of 0.01–100 Hz. The ground electrode was placed at AFz, and electro-oculographic activity was monitored using Ag/AgCl electrodes positioned approximately 1 cm below the left eye. The online reference was CPz in Experiment 1 and FCz in Experiments 2 and 3. Electrode impedances were kept below 5 kΩ.

Preprocessing was performed using EEGLAB [44] with an identical pipeline across experiments to ensure comparability. Data were downsampled to 200 Hz, re-referenced to the average of all scalp electrodes, and filtered using a 50 Hz notch filter and a 0.5–40 Hz band-pass filter. Epochs were extracted from −1000 to 2000 ms relative to stimulus offset for time–frequency analyses.

Independent component analysis (ICA) was applied to identify artifact-related components using the multiple artifact rejection algorithm (MARA; [45]). Components with artifact probabilities exceeding the standard threshold of 80% were removed [45,46]. Epochs containing amplitude fluctuations exceeding ±75 μV were rejected, and participants with more than 20% rejected trials were excluded from EEG analyses. Based on this criterion, one participant from Experiment 1 was excluded.

### 2.4. Analysis

#### 2.4.1. Learning Slope

The primary behavioral measure of rhythm learning was the learning slope, defined as the linear change in the reaction time (RT) difference between random and rhythmic trials across the six-session TR-SRT task. For each participant, the RT difference (RT_random − RT_rhythmic) was regressed on session number to quantify learning progression (e.g., [40]). A positive slope indicates that responses to rhythmic trials became increasingly faster relative to random trials, reflecting acquisition of the temporal rhythm. Using the slope as the learning index captures the overall trajectory of learning across sessions and provides a more robust estimate than RT differences at individual sessions, which may be influenced by session-specific variability [40].

#### 2.4.2. Time-Frequency Magnitude

Time–frequency analyses were performed using the Brainstorm toolbox. Neural activity was decomposed using Morlet wavelets over a time window from −1000 to 2000 ms relative to stimulus offset, with frequencies ranging from 1 to 30 Hz in 0.2-Hz increments. A 1/f spectral compensation procedure was applied to flatten the spectrum and enhance the visibility of oscillatory components. For each frequency, oscillatory magnitude, defined as the square root of power, was log-transformed in decibels (dB) relative to a 2-s post-offset baseline. Thus, zero indicated no power change relative to this baseline. This normalization was used to isolate learning-related power changes.

To identify the dominant frequency band involved in the task, baseline-normalized oscillatory magnitude was first averaged across all EEG channels, sessions, and stimulus types (rhythmic and random) for each participant. This whole-brain (global) measure was used to improve the robustness of theta-band detection by reducing the influence of individual differences in scalp topography and local spectral variability [33,47]. The resulting global spectrum (1–30 Hz) was statistically compared against zero using one-sample *t*-tests across participants. Time–frequency bins (5 ms × 0.2 Hz) within the −1 to 0 s window relative to stimulus offset were considered significantly active if they exceeded an FDR-corrected threshold of *p* < 0.05. This procedure identified significant theta-band activity at 4.8–5.2 Hz within the −0.8 to 0 s window in Experiments 1 and 2 (see Figure S1); this time–frequency range was therefore selected for subsequent analyses. Although activity in the alpha range appeared visually present in the time–frequency plots, it did not survive FDR correction in the pre-offset window (−1000 to 0 ms) and was therefore not considered statistically reliable.

To examine learning-related changes in theta activity, the six TR-SRT sessions were grouped into two stages: early (Sessions 1–3) and late (Sessions 4–6). Theta magnitude was averaged separately for rhythmic and random trials within each stage. A 2 (stimulus type: rhythmic vs. random) × 2 (learning stage: early vs. late) repeated-measures ANOVA was then performed to evaluate the effects of rhythmic structure and learning progression on theta activity.

The same analysis pipeline was applied across all three experiments to ensure methodological consistency.

#### 2.4.3. Awareness Score of Rhythmic Knowledge

To evaluate discrimination within each attribution category in the post-learning test, accuracy scores were compared against the 50% chance level using one-sample Bayesian t-tests. Discrimination accuracy for each attribution type was computed using the correction formula (*Nc* + 0.5)/(*N* + 1), where *Nc* denotes the number of correct responses attributed to that category and *N* denotes the total number of responses in the category. This correction provides a stable estimate of accuracy and reduces bias when the number of responses within a category is small.

#### 2.4.4. Bayes Factor and Robustness Region

Bayes factors (BFs) were computed to quantify evidence for or against behavioral learning and neural oscillatory effects.

For the behavioral learning slope, the prior was derived from previous temporal-sequence learning research using a five-item structured sequence [40], which reported an RT learning slope of approximately 6 ms/session. Accordingly, H1 was modeled using a half-normal prior with a standard deviation (SD) of 6 ms/session, denoted as BHN(0, 6 ms/session). A positive slope (RT_random − RT_rhythmic > 0) was treated as the predicted direction of learning.

For neural oscillatory effects, the prior was defined using the Room-to-Move heuristic [48]. The maximal plausible effect size was estimated from the empirically observed whole-brain theta magnitude during task performance. Because the observed oscillatory magnitude approximates half of the maximal possible condition difference (rhythmic − random), the SD of the half-normal prior was set to this value (0.68 dB), yielding BHN(0, 0.68 dB) for analyses of theta power.

For the structural knowledge test, discrimination accuracy was compared with the 50% chance level using one-sample Bayesian t-tests against chance performance. Because accuracy in implicit-learning awareness tests typically deviates only modestly from chance, the alternative hypothesis was modeled using a half-Cauchy prior based on the Room-to-Move heuristic [48]. The scale parameter was set to 7%, corresponding to the plausible deviation from chance performance (50%/7 ≈ 7%), yielding BHC (0, 7%) for analyses of awareness accuracy across attribution categories (“Guess”, “Intuition”, “Memory”, and “Rule”; [48]).

BFs were interpreted following conventional criteria: B > 3 indicates substantial evidence for an effect, B < 0.33 indicates substantial evidence for the null hypothesis, and 0.33 < B < 3 indicates inconclusive evidence [49].

To evaluate the robustness of conclusions to prior assumptions, robustness regions (RRs) were also reported. RRs indicate the range of prior scale values for which the qualitative BF conclusion remains unchanged [48].

## 3. Results

### 3.1. Learning Slope

Incorrect keypress responses were infrequent across all experiments (Experiments 1–3: 25.08 ± 21.44, 31.28 ± 30.12, and 23.08 ± 17.59 out of 720 trials), yielding mean response accuracies above 95% in each experiment (96.52%, 95.66%, and 96.82%, respectively). All incorrect trials were excluded from analyses.

**Figure 2:**
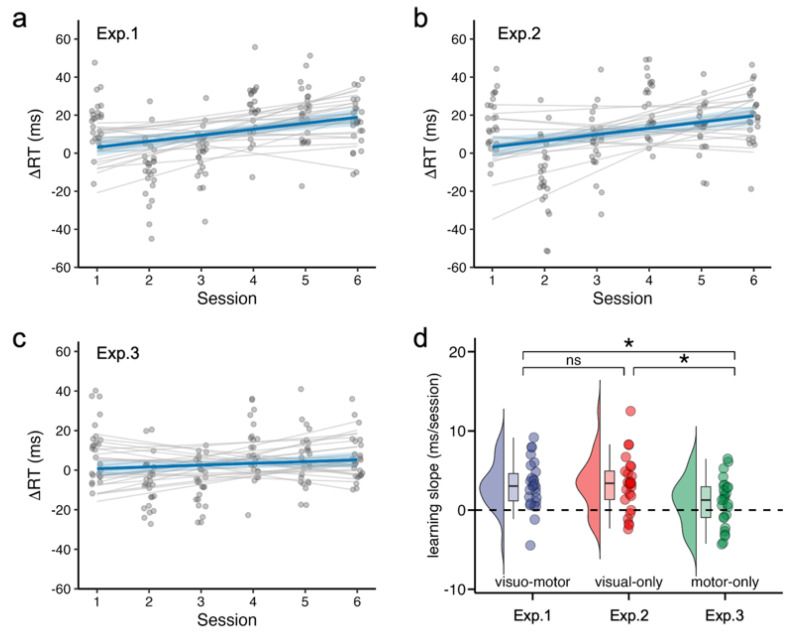
Behavioral evidence of rhythm learning across experiments. (**a**–**c**) Learning trajectories in Experiments 1–3. Each panel shows the reaction–time difference between random and rhythmic trials (ΔRT = RT_random − RT_rhythmic) across the six learning sessions. Positive values indicate faster responses to rhythmic trials relative to random trials. Gray lines represent participant-specific linear fits, and the blue line represents the group-level linear trend with 95% confidence intervals. (**d**) Distribution of individual learning slopes across experiments. Points represent individual participants’ learning slopes (ms/session), with violin and box plots illustrating the distribution and central tendency of the data. Asterisks indicate significant differences between experiments after Bonferroni correction (*p* < 0.05).

Learning slopes are summarized in Table 1. In Experiment 1 (visuo-motor rhythm), the learning slope was significantly greater than zero, indicating robust rhythm learning (Figure 2a). A comparable learning effect was observed in Experiment 2 (visual-only rhythm; Figure 2b). In contrast, Experiment 3 (motor-only rhythm) did not show reliable learning (Figure 2c), and the Bayesian analysis provided only inconclusive evidence.

Cross-experiment comparisons indicated that learning slopes did not differ between Experiments 1 and 2 but were smaller in Experiment 3 (Figure 2d). A one-way ANOVA confirmed a significant effect of experiment on learning slope, *F*(2, 74) = 4.23, *p* = 0.018, *η*_p_^2^ = 0.10.

Bonferroni-corrected post hoc tests showed no difference between Experiments 1 and 2 (mean difference = −0.11 [−1.97, 1.74] ms/session, *p* _Bonferroni_ > 0.999; B_N(0, 6 ms/session)_ = 0.15, RR_B<1/3_ = [2.66, +∞)). In contrast, both Experiments 1 and 2 showed significantly larger learning slopes than Experiment 3 (Exp1 vs. Exp3: mean difference = 2.19 [0.49, 3.88] ms/session, *p* _Bonferroni_ = 0.048; B_N(0, 6 ms/session)_ = 3.77, RR_B>3_ = [0.69, 7.76]; Exp2 vs. Exp3: 2.30 [0.45, 4.15] ms/session, *p*_Bonferroni_ = 0.037; B_N(0, 6 ms/session)_ = 5.33, RR_B>3_ = [0.62, 11.30]).

Together, these results indicate that rhythm learning emerges when a reliable perceptual temporal structure is present (Experiments 1–2), whereas learning is attenuated when periodicity is expressed only in motor timing (Experiment 3).

### 3.2. Theta Power

**Figure 3:**
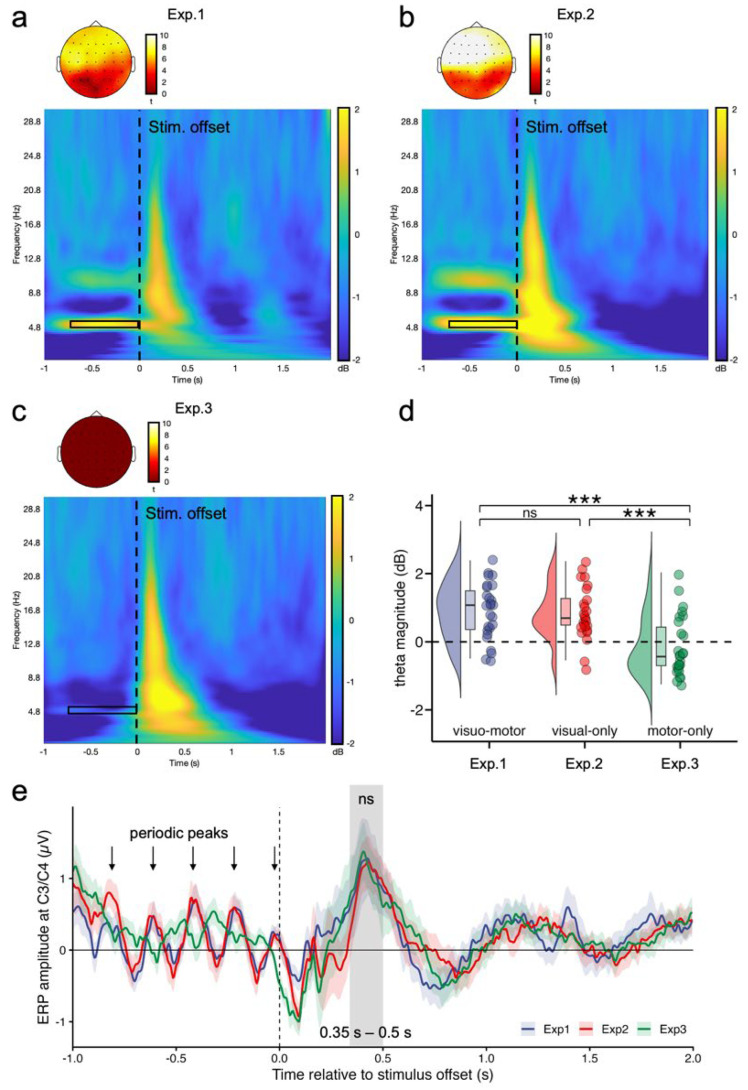
Global theta activity associated with rhythmic temporal structure. (**a**–**c**) Time–frequency representations of global theta activity in Experiments 1–3. The plots show the mean power changes (dB) aligned to stimulus offset (vertical dashed line). The highlighted region (black rectangle) marks the theta band of interest (4.8–5.2 Hz; −800 to 0 ms before stimulus offset) used for statistical analyses. The scalp maps show the t-value distribution from a one-sample *t*-test of theta power averaged within the time–frequency window against zero across participants. The corresponding FDR-corrected time–frequency *t*-maps are provided in Figure S1. (**d**) Distribution of the theta magnitude extracted from the defined time–frequency window for each experiment. Points represent individual participants, and violin and box plots summarize the distribution of theta power (dB). Horizontal dashed lines indicate zero power change relative to the baseline. Asterisks indicate significant between-experiment differences after Bonferroni correction (*** *p* < 0.001; ns = non-significant). (**e**) Grand-average stimulus-offset-locked ERP waveforms over bilateral central electrodes C3/C4 across the three experiments. Shaded areas indicate 95% confidence intervals.

In Experiment 1, significant global theta activity (4.8–5.2 Hz; −800 to 0 ms relative to stimulus offset; Figure 3a and Figure S1) was observed across conditions (1.36 [0.98, 1.73] dB, *t*(25) = 7.48, *p* < 0.001, *d* = 1.47) with decisive Bayesian evidence (B_HN(0, 0.68 dB)_ = 1.09 × 10^11^, RR_B>3_ = [0.03, 1.65×10^11^]). The repeated-measures ANOVA (Table 2) revealed a significant main effect of stimulus type, indicating greater theta magnitude for rhythmic than for random trials. No main effect of learning stage or interaction between stimulus type and learning stage was observed, suggesting that theta enhancement remained stable across early and late learning stages.

Experiment 2 showed a similar pattern (Figure 3b and Figure S1). Robust theta activity was again observed within the same frequency and time window (2.19 [1.78, 2.60] dB, *t*(24) = 10.55, *p* < 0.001, *d* = 2.11), with decisive Bayesian evidence (B_HN(0, 0.68 dB)_ = 7.51 × 10^21^, RR_B>3_ = [0.02, 2.26 × 10^23^]). The ANOVA (Table 2) likewise revealed a significant main effect of stimulus type, reflecting stronger theta magnitude for rhythmic than for random trials, with no evidence for a main effect of learning stage or for the interaction.

In contrast, Experiment 3 (motor-only rhythm) showed no evidence of theta enhancement (Figure 3c and Figure S1). Instead, theta magnitude within the same frequency and time window was significantly below zero (−1.31 [−1.65, −0.96] dB, *t*(25) = −7.49, *p* < 0.001, *d* = −1.47), with Bayesian evidence favoring the absence of positive theta activation (B_HN(0, 0.68 dB)_ = 0.027, RR_B<1/3_ = [0.05, +∞]). The ANOVA (Table 2) revealed no main effects and no interaction.

Across experiments, a one-way ANOVA on the rhythmic-minus-random theta difference revealed a significant effect of experiment, *F*(2, 74) = 13.36, *p* < 0.001, η_p_^2^ = 0.27. Post hoc pairwise comparisons (Figure 3d) showed no difference between Experiments 1 and 2 (mean difference = 0.05 dB, 95% CI = [−0.39, 0.50], *p*_Bonferroni_ > 0.999, B_N(0, 0.68 dB)_ = 0.32, RR_B<1/3_ = [0.64, +∞]), providing evidence for the null hypothesis. In contrast, both Experiments 1 and 2 showed significantly greater theta enhancement than Experiment 3 (*ps* _Bonferroni_ ≤ 0.00014). Specifically, the difference was 1.04 dB [0.57, 1.51] for Experiment 1 vs. 3 (B_N(0, 0.68 dB)_ = 2326.51, RR_B>3_ = [0.09, 1599.08]) and 0.99 dB [0.53, 1.45] for Experiment 2 vs. 3 (B_N(0, 0.68 dB)_ = 998.52, RR_B>3_ = [0.09, 620.64]).

Alongside the global theta-power analysis, we further examined the scalp distribution of theta activity in Experiments 1 and 2 using one-sample *t*-tests against zero. The scalp maps showed consistent topographies across the two experiments, with theta activity most pronounced over frontal and fronto-central electrodes and weaker over posterior sites (Figure 3a,b).

Together, these results indicate that fronto-central theta enhancement (4.8–5.2 Hz; −800 to 0 ms relative to stimulus offset) emerged only when a structured perceptual temporal sequence was present (Experiments 1–2) and was absent when periodicity was confined to motor timing (Experiment 3). This pattern closely mirrors the behavioral learning-slope results, linking theta enhancement to perceptual-structure–dependent facilitation of response timing.

### 3.3. Control Analysis of Motor-Related ERP Activity

To examine whether the observed neural effects were contaminated by button-press activity, we analyzed stimulus-offset-locked ERPs over bilateral central electrodes C3 and C4, where motor-related activity is typically observed [50]. Because participants responded with both hands, C3 and C4 signals were averaged.

The ERPs showed a motor-related peak at 0.35–0.50 s after stimulus offset across the three experiments (Figure 3e). A one-way ANOVA on mean amplitudes within this window revealed no significant differences among Experiments 1–3, *F*(2, 74) = 0.32, *p* = 0.727, η_p_^2^ = 0.01, indicating that button-press activity cannot account for the theta effects. Notably, clear pre-offset periodic ERP peaks were observed in the visuomotor and visual-only conditions, but not in the motor-only condition, mirroring the time–frequency results and suggesting that rhythmic neural responses were linked to perceptual temporal structure rather than motor execution.

### 3.4. Post-Learning Awareness Scores

We next examined whether the acquired structural knowledge of the rhythm was conscious or unconscious.

In Experiment 2, the proportions of trials classified as “Guess”, “Intuition”, “Memory”, and “Rules” were 23.88 ± 19.34%, 36.68 ± 20.08%, 30.96 ± 25.27%, and 8.48 ± 19.57%, respectively.

**Figure 4:**
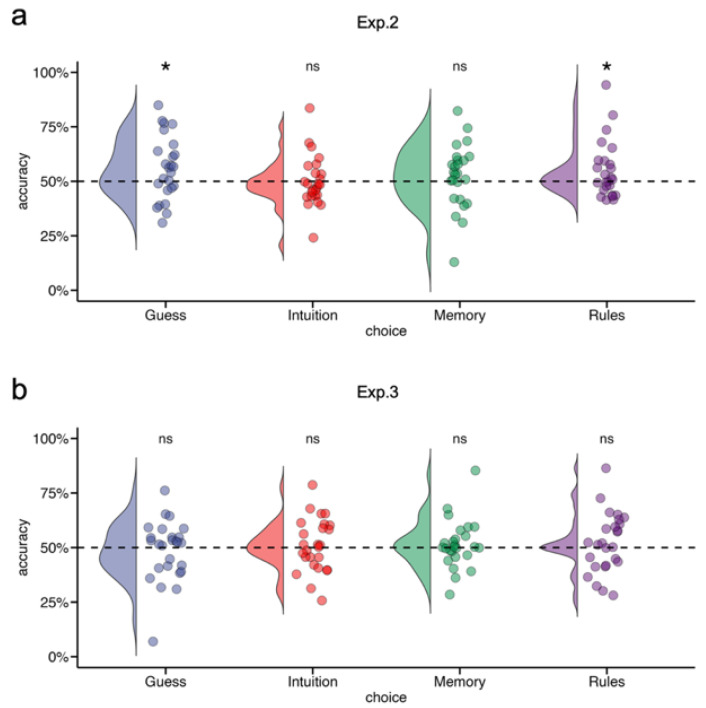
Accuracy of structural knowledge judgments in the post-learning awareness test. (**a**) Experiment 2 (visual-only rhythm) and (**b**) Experiment 3 (motor-only rhythm). Violin plots illustrate the distribution of participants’ discrimination accuracy for the four response attributions (“Guess”, “Intuition”, “Memory”, and “Rules”). Dots represent individual participants. The dashed horizontal line indicates chance level (50%). Asterisks denote significant deviations from chance level based on one-sample *t*-tests (*p* < 0.05; ns = non-significant).

Accuracy exceeded the chance level for responses attributed to both “Guess” and “Rules”, whereas performance based on “Intuition” did not differ from chance. Evidence for “Memory”-based responses was inconclusive (see Table 3 and Figure 4a).

Direct comparison between guess-based and rule-based discrimination accuracy showed no evidence for a difference between the two (*t*(24) = 0.10, *p* = 0.925, Cohen’s *d* = 0.02, B_HC(0, 7%)_ = 0.34, RR_1/3<B<3_ = [0%, 7.36%]), indicating that rhythmic structure could be recognized through both implicit (“Guess”) and explicit (“Rules”) judgments.

To determine which type of knowledge predominated, we compared the knowledge proportion between guess-based and rule-based responses. Knowledge proportion was defined as the proportion of correctly discriminated trials attributed to a given response type relative to the total number of correct trials [27].

This analysis revealed a significantly higher proportion of guess-based than rule-based knowledge (*t*(24) = 2.40, *p* = 0.025, Cohen’s *d* = 0.48, B_HC(0, 7%)_ = 6.13, RR_B>3_ = [1.41%, 56.42%]). Implicit guesses accounted for 24.26% ± 19.23% of correct trials compared with 9.10% ± 19.06% for explicit rules. These results indicate that structural knowledge acquired in the TR-SRT task was predominantly implicit.

The structural knowledge test in Experiment 3 provided no evidence that rhythmic knowledge had been acquired. The proportions of trials classified as “Guess”, “Intuition”, “Memory”, and “Rules” were 24.27 ± 18.72%, 36.08 ± 16.26%, 26.85 ± 20.58%, and 12.81 ± 16.76%, respectively.

Accuracy for the two implicit response categories (“Guess” and “Intuition”) did not exceed the chance level. For the two explicit categories (“Memory” and “Rules”), the evidence for above-chance performance was inconclusive (see Table 3; Figure 4b).

Consistent with the analysis in Experiment 2, discrimination accuracy did not differ between the guess-based and rule-based responses (*t*(25) = −1.01, *p* = 0.320, Cohen’s *d* = −0.20, B_HC(0, 7%)_ = 0.19, RR_B<1/3_ = [3.43%, 50%]).

We further examined the knowledge proportion for guess-based and rule-based responses. Although the proportion of guess-based knowledge (23.27% ± 18.50%) was numerically higher than that of rule-based knowledge (12.54% ± 15.89%), the difference did not reach significance (*t*(24) = 1.80, *p* = 0.084, Cohen’s *d* = 0.35, B_HC(0, 7%)_ = 2.35, RR_1/3<B<3_ = (0%, 100%)).

Together, these results indicate that structural knowledge of the rhythm emerged in the perceptual-rhythm condition and was predominantly implicit (Experiment 2), whereas no reliable structural knowledge was observed when rhythmicity was restricted to motor timing (Experiment 3).

## 4. Discussion

In the present study, we developed a modified serial reaction time task (TR-SRT) that dissociates perceptual and motor temporal structures embedded in visual stimuli and keystrokes. Using this paradigm, Experiment 1 employed synchronized perceptual and motor rhythms and revealed robust rhythm learning. Experiment 2 disrupted the motor rhythm while preserving the perceptual rhythm, yet learning remained comparable to Experiment 1 and was largely implicit. In contrast, Experiment 3 disrupted the perceptual rhythm while maintaining motor rhythmicity, and no reliable learning was observed. Together, these findings indicate that rhythm learning in dynamic environments primarily depends on perceptual temporal input rather than on the stereotyped repetition of actions within the SRT framework.

Our findings demonstrate that implicit rhythm knowledge can be acquired through perceptual input, consistent with previous evidence for the implicit learning of periodic structures in visual sequences [25,51]. In standard SRT tasks, however, the synchrony between action execution and perceptual input makes it difficult to dissociate the respective contributions of perceptual encoding and motor production. By separating these two components, the present study shows that continuous perceptual rhythmic input plays a dominant role in rhythm learning, whereas motor rhythmicity alone is insufficient to support learning. This result suggests that perceptual timing mechanisms—such as internal temporal representations or predictive timing processes—are central to the acquisition of rhythmic knowledge. Within this framework, rhythmic actions may reflect behavioral outputs guided by perceptually derived temporal representations rather than constituting the primary source of rhythm learning.

Theta oscillations have been implicated in the formation, updating, and integration of knowledge representations [31,32,52,53]. Evidence from both humans and non-human primates further suggests that theta activity can emerge during learning, even in the absence of conscious awareness [27,35,36,54], and its magnitude has been linked to implicit learning efficiency [37]. Consistent with these findings, our EEG results showed increased global theta magnitude when rhythm learning occurred in Experiments 1 and 2, whereas no such enhancement was observed when learning was absent in Experiment 3. The use of global theta magnitude provided a robust summary measure by reducing the sensitivity to individual differences in EEG topography and local spectral variability. This approach is particularly suitable for theta activity, which often reflects distributed neural coordination rather than activity from a single focal source and can propagate across long-range networks during learning and memory [33,47]. These findings suggest that global theta power may serve as a neural index of rhythmic knowledge acquisition.

The scalp maps further indicated that theta power was most pronounced over frontal and fronto-central electrodes. Frontal theta is thought to support learning-related prediction and control [55,56] and to coordinate communication with other regions, including the hippocampus [57]. Recent evidence suggests that theta activity can propagate from posterior to anterior regions during successful memory encoding [58]. This fronto-central pattern is therefore consistent with the view that theta activity may support the encoding and prediction of perceptual temporal structure.

The TR-SRT task also provides methodological advantages for studying temporal learning. By reducing rhythmic patterns that arise from sequential motor execution—including stimulus-evoked saccades—it allows researchers to more directly examine the role of perceptual temporal processing. The temporal structure embedded in the TR-SRT paradigm can be flexibly manipulated, for example, by varying the cycle length or rhythmic complexity, enabling the systematic investigation of different aspects of temporal learning. Future work could also extend this paradigm to auditory presentation to examine cross-modal rhythm learning. More broadly, successful interaction with dynamic environments requires sensitivity to temporal regularities, and the present findings suggest that such regularities can be detected and learned implicitly through perceptual processing.

Although the present findings provide evidence that perceptual temporal input plays a dominant role in rhythm learning, several directions remain for future research. First, the present study focused on visual rhythmic structures within a modified SRT paradigm. Previous work suggests that auditory temporal sequences may be processed with higher sensitivity than visual ones when perceptual and motor components are not dissociated [10], leaving open the question of whether the perceptual dominance observed here would generalize to auditory or multisensory contexts. Future studies could extend the TR-SRT paradigm to auditory or multimodal presentations to test whether perceptual temporal input consistently plays the dominant role across modalities. Second, because the present study used scalp EEG, the observed topographical pattern should be interpreted at the scalp level rather than as evidence for specific neural generators. Future studies using source-resolved MEG, intracranial EEG, or causal brain stimulation could further clarify the cortical distribution and directional transfer of theta activity during rhythm learning.

## 5. Conclusions

The present study introduced a modified serial reaction time paradigm (TR-SRT) that dissociates perceptual and motor temporal structures, allowing their independent contributions to rhythm learning to be examined. Across three experiments, behavioral results showed that rhythm learning emerged when perceptual rhythmic input was preserved, regardless of motor rhythmicity, whereas motor rhythms alone did not support learning. Post-learning awareness measures further indicated that the acquired rhythmic knowledge was predominantly implicit. EEG analyses revealed that learning was accompanied by enhanced theta activity, providing a neural index of rhythmic knowledge acquisition. Together, these findings indicate that the learning of temporal regularities in dynamic environments is primarily driven by perceptual temporal input, highlighting perceptual timing mechanisms as a key foundation for implicit rhythm learning.

## Figures and Tables

**Table 1:** Behavioral learning slopes across experiments in the TR-SRT task.

Experiment	Mean Slope (ms/Session)	95% CI	*t* (df)	*p*	*d*	B_HN(0, 6 ms/Session)_	Bayesian Evidence	Robustness Region
Exp1 (Visuo-motor)	3.15	[1.92, 4.38]	5.28 (25)	<0.001	1.04	2.00 × 10^5^	B > 3	[0.12, 4.60 × 10^5^]
Exp2 (Visual-only)	3.27	[1.81, 4.72]	4.64 (24)	<0.001	0.93	9672.95	B > 3	[0.17, 2.30 × 10^4^]
Exp3 (Motor-only)	0.97	[−0.26, 2.19]	1.63 (25)	0.116	0.32	0.69	1/3 < B < 3	[0, 12.77]

Note. Learning slopes reflect the linear change in the reaction-time (RT) difference between random and rhythmic trials across the six TR-SRT sessions. Positive slopes indicate progressively faster responses to rhythmic trials relative to random trials, reflecting rhythm learning. Bayesian evidence was evaluated using a half-normal prior with SD = 6 ms/session (BHN(0, 6 ms/session)). Robustness regions indicate the range of prior scale values for which the qualitative Bayes factor conclusion remains unchanged. Evidence categories follow conventional criteria: B > 3 indicates evidence for learning, B < 0.33 indicates evidence for the absence of learning, and 0.33 < B < 3 indicates inconclusive evidence.

**Table 2:** Theta-band power effects across conditions in Experiments 1–3.

Experiment	Condition	Mean	95% CI	*F* (df1, df2)	*p*	η_p_^2^	B_HN(0, 0.68 dB)_	Bayesian Evidence	Robustness Region
Exp.1	Rhythmic	1.81	[1.36, 2.25]	15.25 (1, 25)	0.0002	0.38	1.38 × 10^6^	B > 3	[0.03, 7.35 × 10^5^]
	Random	0.91	[0.46, 1.35]						
	Early	1.43	[0.99, 1.86]	0.38 (1, 25)	0.541	0.01	0.53	1/3 < B < 3	[0, 1.13]
	Late	1.29	[0.77, 1.80]						
	Interaction	—	—	0.09 (1, 25)	0.765	0.004	0.55	1/3 < B < 3	[0, 1.21]
Exp.2	Rhythmic	2.61	[2.13, 3.10]	12.53 (1, 24)	0.0006	0.34	1.17 × 10^6^	B > 3	[0.03, 5.70 × 10^5^]
	Random	1.77	[1.27, 2.26]						
	Early	2.16	[1.63, 2.68]	0.06 (1, 24)	0.802	0.003	0.19	B < 1/3	[0.37, +∞)
	Late	2.22	[1.72, 2.72]						
	Interaction	—	—	0.03 (1, 24)	0.875	0.001	0.51	1/3 < B < 3	[0, 1.14]
Exp.3	Rhythmic	−1.38	[−1.85, −0.90]	0.37 (1, 25)	0.544	0.01	0.14	B < 1/3	[0.26, +∞)
	Random	−1.23	[−1.69, −0.77]						
	Early	−1.41	[−1.88, −0.94]	0.84 (1, 25)	0.361	0.03	0.13	B < 1/3	[0.25, +∞)
	Late	−1.20	[−1.66, −0.73 ]						
	Interaction	—	—	0.01 (1, 25)	0.905	0.001	0.99	1/3 < B < 3	[0, 2.64]

Note. Mean theta power (dB) and 95% confidence intervals (95% CI) are reported for each condition (rhythmic vs. random; early vs. late) across Experiments 1–3. Theta power was averaged within the defined time–frequency window (4.8–5.2 Hz; −800 to 0 ms relative to stimulus offset). Bayesian evidence was quantified using Bayes factors (B), where B > 3 indicates evidence for the alternative hypothesis, B < 1/3 indicates evidence for the null hypothesis, and values between 1/3 and 3 indicate inconclusive evidence. B_HN_ represents the Bayes factor computed under the half normal prior (0, 0.68 dB). Robustness regions indicate the range of prior scales over which the qualitative Bayesian inference remains stable.

**Table 3:** Structural knowledge awareness performance in Experiments 2 and 3.

	Choice	Mean (%)	95% CI	*t* (df)	*p*	*d*	B_HC(0, 7%)_	Bayesian Evidence	Robustness Region (%)
Exp.2	Guess	55.52	[50.86, 60.19]	2.32 (24)	0.029	0.46	4.69	B > 3	[0.70, 15.22]
	Intuition	49.22	[45.18, 53.27]	−0.38 (24)	0.711	−0.08	0.17	B < 1/3	[3.13, 50]
	Memory	52.29	[47.11, 57.47]	0.87 (24)	0.395	0.17	0.58	1/3 < B < 3	[0, 14.18]
	Rules	55.21	[50.77, 59.65]	2.30 (24)	0.030	0.46	4.34	B > 3	[0.73, 13.31]
Exp.3	Guess	48.33	[43.65, 53.01]	−0.70 (25)	0.491	−0.14	0.16	B < 1/3	[2.83, 50]
	Intuition	50.48	[46.71, 54.24]	0.25 (25)	0.806	0.05	0.25	B < 1/3	[5.02, 50]
	Memory	51.65	[47.43, 55.86]	0.77 (25)	0.450	0.15	0.44	1/3 < B < 3	[0, 9.78]
	Rules	51.86	[46.63, 57.09]	0.70 (25)	0.492	0.14	0.50	1/3 < B < 3	[0, 11.70].

Note. Mean accuracy (%) and 95% confidence intervals (95% CI) are reported for each response attribution (“Guess”, “Intuition”, “Memory”, and “Rules”) in Experiments 2 and 3. One-sample t-tests were conducted against chance level (50%). Effect sizes are reported as Cohen’s d. Bayesian evidence was quantified using Bayes factors (B), where B > 3 indicates evidence for above-chance performance (alternative hypothesis), B < 1/3 indicates evidence for chance-level performance (null hypothesis), and values between 1/3 and 3 indicate inconclusive evidence. B_HC_ denotes the Bayes factor under the default prior (0, 7%). Robustness regions indicate the range of prior scales over which the qualitative Bayesian inference remains stable.

## Data Availability

All data, analysis codes, and experimental materials of this study are available via the Open Science Framework, Experiment 1: https://osf.io/wjvf6/ (accessed on 27 April 2026); Experiment 2: https://osf.io/24ebu/ (accessed on 27 April 2026); Experiment 3: https://osf.io/j3sw9/ (accessed on 27 April 2026).

## Supplementary Materials

The following supporting information can be downloaded at: https://www.mdpi.com/article/10.3390/brainsci16050489/s1; Figure S1: Theta-specific activation revealed by time–frequency statistical analysis. A reference [59] is cited in the Supplementary Materials.
