# Supplementary material for: Perceptual Temporal Structure Supports Rhythm Learning and Enhances Theta Oscillations When Perception and Action Are Dissociated
Source: Brain Sci. 2026 Apr 30;16(5):489. doi: 10.3390/brainsci16050489 (PMC13204124; doi:10.3390/brainsci16050489)
Supplement: Supplementary file 1 [file brainsci-16-00489-s001.zip › brainsci-4235217-supplementary.pdf]

Perceptual Temporal Structure Supports Rhythm Learning and Enhances Theta Oscillations When Perception and Action Are Dissociated

Supplementary Materials

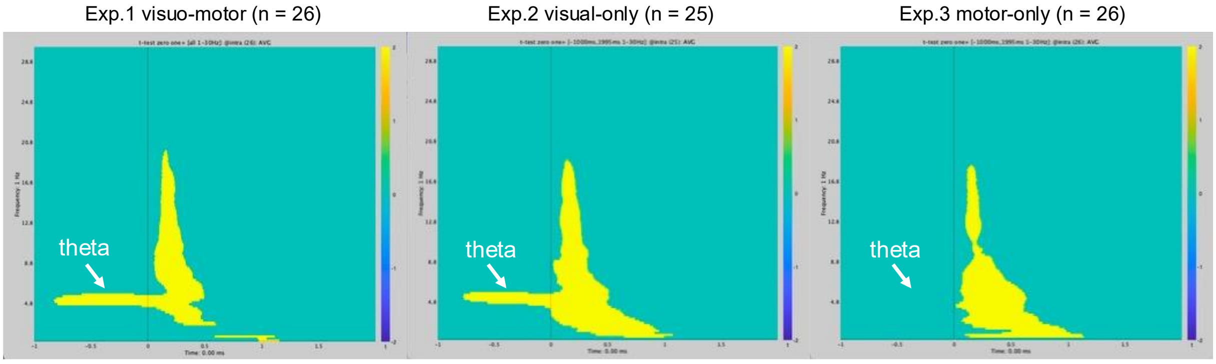

Figure S1. Theta-specific activation revealed by time–frequency statistical analysis. Time–frequency representations of global oscillatory activity in Experiments 1–3, with statistical significance overlaid. Oscillatory magnitude (square root of power) was baseline-normalized relative to the post-offset interval (0–2 s) and compared against zero using one-sample *t*-tests across participants. Statistical significance was determined for each time–frequency bin (5 ms × 0.2 Hz) with false discovery rate (FDR) correction applied across both time and frequency dimensions ($p < 0.05$). Significant activation was observed selectively in the theta band (4.8–5.2 Hz) during the pre-offset interval (approximately −0.8 to 0 s) in Experiments 1 and 2. In contrast, although activity in the alpha range (8–12 Hz) appeared visually present (Figure 3a, 3b), it did not survive FDR correction and was therefore not considered statistically reliable. The apparent alpha-band activity likely reflects harmonic components of the dominant theta oscillation. Given a fundamental frequency around 5 Hz, integer harmonics (e.g., ~10 Hz) may emerge in time–frequency representations due to the non-sinusoidal structure of neural oscillations (e.g.,[59]).This pattern indicates that the observed neural effect is specific to theta-band activity rather than reflecting independent alpha-band processes.
